# Novel *Odoribacter splanchnicus* Strain and Its Outer Membrane Vesicles Exert Immunoregulatory Effects *in vitro*

**DOI:** 10.3389/fmicb.2020.575455

**Published:** 2020-11-12

**Authors:** Kaisa Hiippala, Gonçalo Barreto, Claudia Burrello, Angelica Diaz-Basabe, Maiju Suutarinen, Veera Kainulainen, Jolene R. Bowers, Darrin Lemmer, David M. Engelthaler, Kari K. Eklund, Federica Facciotti, Reetta Satokari

**Affiliations:** ^1^Human Microbiome Research Program, Faculty of Medicine, University of Helsinki, Helsinki, Finland; ^2^Translational Immunology Research Program, Faculty of Medicine, University of Helsinki, Helsinki, Finland; ^3^Department of Experimental Oncology, European Institute of Oncology, Milan, Italy; ^4^Translational Genomics Research Institute, Pathogen and Microbiome Division, Flagstaff, Arizona, AZ, United States; ^5^Helsinki University and Helsinki University Hospital, Department of Rheumatology, Helsinki, Finland and ORTON Orthopedic Hospital of the Orton Foundation, Helsinki, Finland

**Keywords:** Odoribacter, LPS, OMV, host-microbe interactions, immunoregulation, gut microbiota

## Abstract

*Odoribacter splanchnicus*, belonging to the order Bacteroidales, is a common, short-chain fatty acid producing member of the human intestinal microbiota. A decreased abundance of *Odoribacter* has been linked to different microbiota-associated diseases, such as non-alcoholic fatty liver disease, cystic fibrosis and inflammatory bowel disease (IBD). The type strain of *O. splanchnicus* has been genome-sequenced, but otherwise very little is known about this anaerobic bacterium. The species surfaces in many microbiota studies and, consequently, comprehension on its interactions with the host is needed. In this study, we isolated a novel strain of *O. splanchnicus* from a healthy fecal donor, identified it by genome sequencing and addressed its adhesive, epithelium reinforcing and immunoregulatory properties. Our results show that *O. splanchnicus* strain 57 is non-adherent to enterocytes or mucus, does not reinforce nor compromise Caco-2 monolayer integrity and most likely harbors penta-acylated, less endotoxic lipid A as part of its lipopolysaccharide (LPS) structure based on the lack of gene *lpxM* and *in vitro* results on low-level NF-κB activity. The studies by transmission electron microscopy revealed that *O. splanchnicus* produces outer membrane vesicles (OMV). *O. splanchnicus* cells, culture supernatant i.e., spent medium or OMVs did not induce interleukin-8 (IL-8) response in HT-29 enterocyte cells suggesting a very low proinflammatory capacity. On the contrary, the treatment of HT-29 cells with *O. splanchnicus* cells, spent medium or OMVs prior to exposure to *Escherichia coli* LPS elicited a significant decrease in IL-8 production as compared to *E. coli* LPS treatment alone. Moreover, *O. splanchnicus* spent supernatant induced IL-10 production by immune cells, suggesting anti-inflammatory activity. Our *in vitro* findings indicate that *O. splanchnicus* and its effector molecules transported in OMVs could potentially exert anti-inflammatory action in the gut epithelium. Taken together, *O. splanchnicus* seems to be a commensal with a primarily beneficial interaction with the host.

## Introduction

*Odoribacter splanchnicus* type strain 1651/6^*T*^, previously classified as *Bacteroides splanchnicus*, was originally isolated from a human stool specimen and abdominal abscess (Werner et al., 1975). In 2008, the new genus *Odoribacter* was separated from *Bacteroides* forming its own cluster within the order Bacteroidales and family Odoribacteraceae (Hardham et al., 2008; Munoz et al., 2016). The two other species belonging to the genus are *Odoribacter laneus*, isolated from human feces (Nagai et al., 2010), and *Odoribacter denticanis*, isolated from canine periodontitis (Hardham et al., 2008). Gram-negative, strictly anaerobic and non-spore forming *O. splanchnicus* is a common member of human intestinal microbiota (Göker et al., 2011). The complete genome sequence of the type strain revealed that only 61% of the protein-coding genes could be associated with a putative function (Göker et al., 2011). The growth of *O. splanchnicus* is enhanced by hemin and bile acid. Its main fermentation products are acetic, propionic and succinic acids, but the bacterium also produces butyric, isovaleric and isobutyric acids in smaller quantities (Werner et al., 1975). In fact, the beneficial effects of *Odoribacter* as part of a healthy, balanced human gut microbiota are primarily attributed to its capacity to produce short-chain fatty acids (SCFA). Dietary fiber and resistant starch are metabolized by commensal bacteria to SCFAs which in turn serve as a vital energy source for the intestinal epithelial cells (Koh et al., 2016). These bacterial metabolites, especially butyrate, have a wide range of health-promoting effects on the gut epithelium, such as maintaining epithelial barrier function and gut homeostasis, exerting anti-inflammatory and immunomodulatory activity in the intestinal mucosa as well as suppressing proinflammatory cytokines (Parada Venegas et al., 2019).

The lower relative abundance of *Odoribacter* has been associated with different disorders, such as non-alcoholic fatty liver disease (NAFLD), cystic fibrosis and inflammatory bowel disease (IBD) (Morgan et al., 2012; Lewis et al., 2015; Burke et al., 2017; Li et al., 2018; Wang et al., 2018). The abundance of *Odoribacter* was found to be reduced in the microbiota of patients with ileal Crohn’s disease (CD) and pancolitis, which is the most severe form of ulcerative colitis (UC) affecting the entire colon (Morgan et al., 2012). Pediatric CD patients had also reduced relative abundance of *Odoribacter* compared to the healthy individuals (Lewis et al., 2015; Wang et al., 2018). The levels of *Odoribacter* and other SCFA-producing gut bacteria were elevated and maintained after Infliximab therapy in pediatric CD patients compared to the controls, suggesting that a sustained response to the therapy was associated with the abundance of SCFA-producing taxa in the gut (Wang et al., 2018). Furthermore, diarrhea-predominant irritable bowel syndrome (IBS) patients with a higher relative abundance of *Odoribacter* and *Faecalibacterium* at baseline level were responding better to a multispecies probiotic combination treatment, having reduction in symptoms and beneficial changes in inflammatory markers (Hod et al., 2018).

The role of *Odoribacter* abundance and the availability of SCFAs in metabolic disorders still remains an open question. The abundance of genus *Odoribacter* was negatively correlated with systolic blood pressure in obese and overweight pregnant women, suggesting that SCFA-producing taxa could have an influence on the host blood pressure (Gomez-Arango et al., 2016). Interestingly, *Odoribacter* seems to be associated with metabolic health benefits coupled with *Akkermansia* based on previous studies on metabolism-related pathologies, such as obesity, metabolic syndrome and diabetes (Brahe et al., 2015; Etxeberria et al., 2017; Lim et al., 2017; Lai et al., 2018). In a twin study, SCFA-producing genera *Akkermansia* and *Odoribacter* were enriched in healthy individuals compared to adults with metabolic syndrome (Lim et al., 2017). Furthermore, these two genera positively correlated with a healthy lipid metabolism profile in obese women (Brahe et al., 2015). On the contrary, one study showed a higher prevalence of *Odoribacter* in male subjects with hypercholesterolemia (Granado-Serrano et al., 2019). In a genetic murine model of obesity, both *Akkermansia* and *Odoribacter* were increased during a Pterostilbene treatment, which has been shown to improve metabolic profile, such as insulin sensitivity (Etxeberria et al., 2017). In addition, *Odoribacter* was associated with the beneficial effect of fecal microbiota transplantation (FMT) in a mouse model regardless of the diet, as the relative abundance of the bacterium increased as a result of FMT in high fat and normal diet groups (Lai et al., 2018).

Besides SCFAs, *O. splanchnicus* has been shown to produce specific bacterial sphingolipids i.e., sulfonolipids, which potentially exert a bioactive function in the intestine (Walker et al., 2017). Sphingolipids, produced by eukaryotes and a small subset of bacterial species, and their metabolites can regulate various cellular processes in the host, such as cell proliferation, apoptosis, differentiation and inflammation (Heaver et al., 2018). *Bacteroides*-derived sphingolipids participate in maintaining gut homeostasis as shown in a study where germ-free mice colonized with sphingolipid-deficient *B. thetaiotaomicron* developed intestinal inflammation and altered ceramide pools (Brown et al., 2019). Sphingolipids and other bacterial molecules are likely transported in outer membrane vesicles (OMV), which are spherical, membranous blebs released from the outer membrane of Gram-negative bacteria, and able to navigate through the thick mucus layer to the epithelium (Schwechheimer and Kuehn, 2015; Heaver et al., 2018). Indeed, OMVs produced by *Bacteroides* spp. have been localized in the intestinal mucosa interacting with the host immune cells (Hickey et al., 2015; Maerz et al., 2018).

The studies on the host-microbe interactions of *O. splanchnicus* are scarce creating a clear need to investigate its properties. In this study, we addressed the adhesive, epithelium reinforcing and immunomodulatory properties of *O. splanchnicus* strain isolated from a healthy fecal donor. We also studied the effect of *O. splanchnicus* cells, LPS, spent medium and OMVs on inflammatory responses in different human cells. To our knowledge, this is the first study to visualize *O. splanchnicus* OMVs and to study their anti-inflammatory potential.

## Results

### Genome of the Strain *O. splanchnicus* 57

The genome of novel *O. splanchnicus* 57 strain (isolated from a healthy fecal donor) was obtained by whole genome sequencing (WGS) to accurately identify the bacterium on species level with Kraken (Wood and Salzberg, 2014). The assembled genome size was 4.2 Mb with a G + C content of 43.3% (Table 1). Of the 3544 genes, 1687 (47.6%) were hypothetical proteins without a putative function assigned. *O. splanchnicus* 57 genome assembly matched the *O. splanchnicus* type strain 1651/6^*T*^ genome (NC_015160.1) with 99.84% identity and 85% query coverage. The assembled genome data of *O. splanchnicus* 57 was screened for genes that encode enzymes for Kdo2-lipid A modification using BLAST (Basic Local Alignment Search Tool, BLASTn algorithm). The genes encoding LpxA-LpxL acyltransferases for the Kdo2-lipid A moiety synthesis were found, but the genome lacked the gene *lpxM* needed for the hexa-acylation of the lipid A, which indicates that *O. splanchnicus* 57 is likely to produce less toxic, penta-acylated form of LPS.

**TABLE 1:** Genome properties of *O. splanchnicus* 57.

**Feature**	**Value**	**% of total**
Genome size (bp)	4,177,878	100%
DNA G + C content (bp)	1,809,021	43.3%
Total genes	3544	100%
Coding sequences (CDS)	3491	98.5%
Hypothetical proteins	1687	47.6%
Transfer RNA genes	52	1.7%

### Mucosal Adherence and Epithelium Reinforcing Properties

**FIGURE 1:**
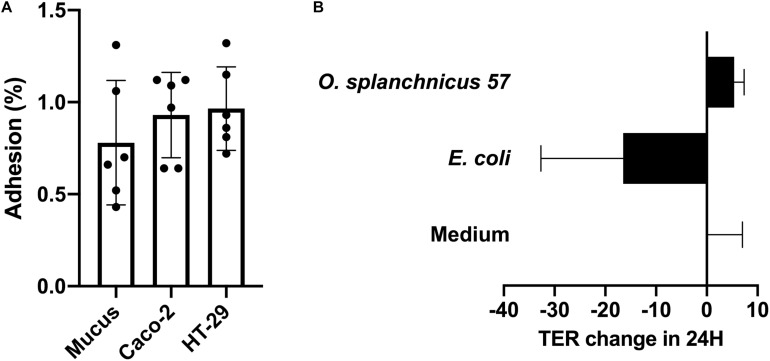
**(A)** Adherence (%) of *O. splanchnicus* 57 to 8-day-old Caco-2 and HT-29 cell lines as well as mucus. Adherence-% reflects the proportion of bound bacteria from that of the added. Data is shown as a mean and standard deviation of six technical replicates. **(B)** The effect of *O. splanchnicus* 57 cells on TER of 8-day-old Caco-2 monolayer. Results are shown as the mean of TER value change (Ω/cm^2^) in 24 h and standard deviation of three technical replicates (parallel wells). *E. coli* K12 was used as negative control and RPMI 1640 as the medium control. In both panels **(A,B)** results are from a representative experiment.

We studied the adherence of *O. splanchnicus* 57 to (8 days post-plating) Caco-2 and HT-29 cell lines as well as to porcine mucus. The relative adherence percentage of the bacterium to the enterocyte cell lines and mucus was below 1%, which is considered to be unspecific, background level binding (Vesterlund et al., 2006; Figure 1A). Thus, *O. splanchnicus* 57 can be considered as non-adherent. In addition, we studied the ability of the isolate to enhance Caco-2 monolayer integrity using transepithelial electrical resistance (TER) measurements (Figure 1B). *O. splanchnicus* 57 did not induce changes in the epithelial integrity as the change in TER values remained at the same level as the medium control indicating that the isolate did not reinforce or compromise the epithelial monolayer.

### Stimulation of PBMCs With *O. splanchnicus* 57 Cells, Spent Medium and LPS

**FIGURE 2:**
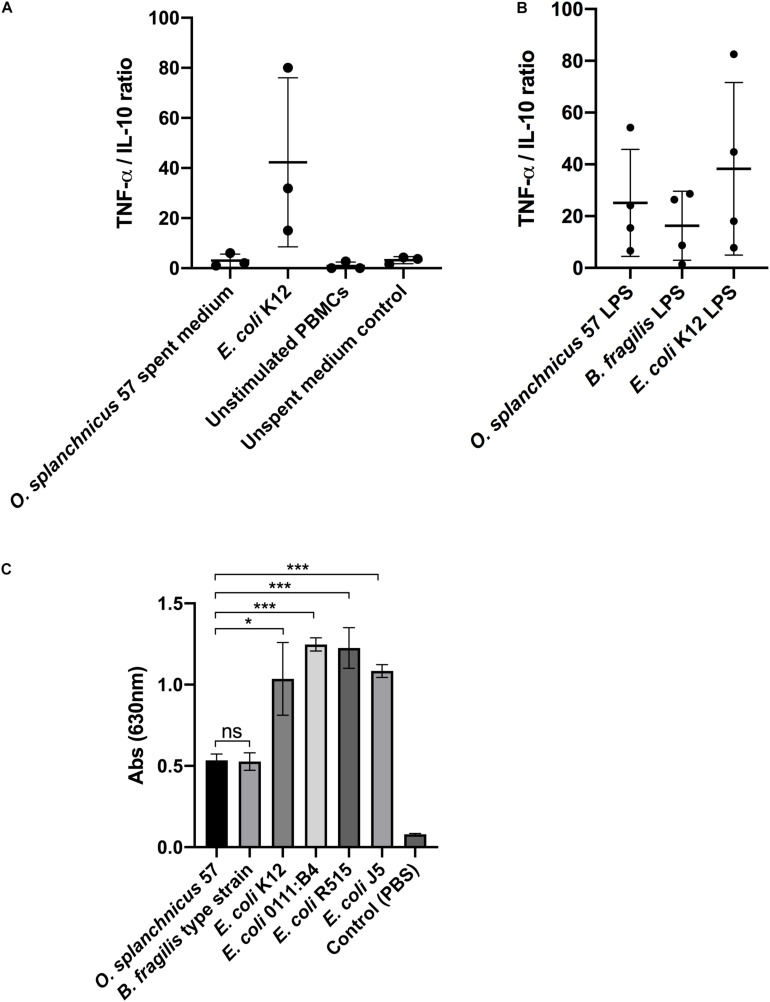
**(A)** Ratio of TNF-α and IL-10 released from PBMCs after stimulation with *O. splanchnicus* 57 spent medium. Heat-killed *E. coli* K12, unstimulated PBMCs and unspent medium were used as controls. The results are presented as a mean and standard deviation of three biological experiments using PBMCs isolated from the buffy coats of three different donors. **(B)** The ratio of TNF-α/IL-10 released from PBMCs after stimulation with 1 ng/ml of *O. splanchnicus* 57 and *B. fragilis* type strain LPS. *E. coli* K12 LPS was used as a control. The results are presented as a mean and standard deviation of four biological experiments using PBMCs isolated from the buffy coats of four different donors. **(C)** Activation of NF-kB in human THP1 dual reporter cells by stimulation with 10 ng/ml LPS extracted from *O. splanchnicus* 57, *B. fragilis* type strain and *E. coli* K12. LPS from three other commercial *E. coli* strains, as well as PBS, were used as controls.

Next, we examined the immunomodulatory effect of *O. splanchnicus* 57 cells and the culture supernatant, i.e., spent medium, on the TNF-α and IL-10 production by healthy human peripheral blood mononuclear cells (PBMCs). Heat-killed *E. coli* K12 was used as a proinflammatory control stimulus in both experiments. There was no significant difference in the TNF-α/IL-10 ratio released from PBMCs following a 24-h stimulation with *O. splanchnicus* 57 cells or *E. coli* K12 (Data not shown). However, the spent growth medium of *O. splanchnicus* 57 induced significantly higher levels of IL-10 in relation to TNF-α compared to *E. coli* K12 suggesting different amounts of pro- and anti-inflammatory bacterial effector molecules present in the spent medium as compared to intact bacterial cells (Figure 2A; Supplementary Figure 1). TNF-α and IL-10 production by PBMCs is presented as ratios collectively from all donors in Figure 2A and as total concentrations from each donor separately in Supplementary Figure 1. PBMCs from each individual donor produced significantly higher IL-10 and lower TNF-α levels after a stimulation with *O. splanchnicus* 57 spent medium compared to *E. coli* (Supplementary Figure 1). The *O. splanchnicus* 57 spent medium stimulated IL-10 release in human PBMCs ranging between 100–330 pg/ml depending on the donor (Supplementary Figure 1). The GAM growth medium for *O. splanchnicus* 57 itself (i.e., unspent medium control) did not affect the cytokine production in PBMCs. Stimulation of human PBMCs with LPS isolated from *O. splanchnicus* 57 and *E. coli* K12 showed no significant difference in the ratio of proinflammatory cytokine TNF-α to anti-inflammatory cytokine IL-10 measured after 24 h of stimulation (Figure 2B). The ratio of TNF-α/IL-10 produced by PBMCs was lower but not statistically significant after induction with *O. splanchnicus* 57 or *B. fragilis* type strain LPS as compared to *E. coli* K12 LPS. While *B. fragilis* type strain LPS stimulated statistically higher IL-10 production in PBMCs from all donors, *O. splanchnicus* 57 LPS stimulated higher IL-10 production in PBMCs of one out of four donors as compared to *E. coli* LPS (Supplementary Figure 2).

### *O. splanchnicus* 57 LPS-Induced NF-κB Signaling in THP-1 Cells

The lack of gene *lpxM* in the *O. splanchnicus* 57 genome indicated that the bacterium potentially harbors a penta-acylated lipid A, making its LPS less proinflammatory as compared to hexa-acylated LPS of *E. coli*. LPS extract from *O. splanchnicus* 57 was used to stimulate human THP-1 dual reporter cells to study the induction of NF-**κ**B signaling (Figure 2C). After a 24-h stimulation, the level of NF-**κ**B activity induced by *O. splanchnicus* 57 LPS was equivalent to *B. fragilis* type strain that was used as control (Figure 2C). The lipid A of *B. fragilis* type strain LPS has a penta-acylated, less endotoxic form compared to *E. coli* LPS (Weintraub et al., 1989), as also shown by the level of NF-**κ**B signaling in human THP-1 cells induced by different bacterial LPSs in our experiments. Taken together, the results suggest that *O. splanchnicus* 57 LPS is less pro-inflammatory as compared to *E. coli* LPS and that the spent medium of *O. splanchnicus* 57 contains substances that counteract the pro-inflammatory effect of LPS.

### *O. splanchnicus* 57 Secreted Outer Membrane Vesicles

**FIGURE 3:**
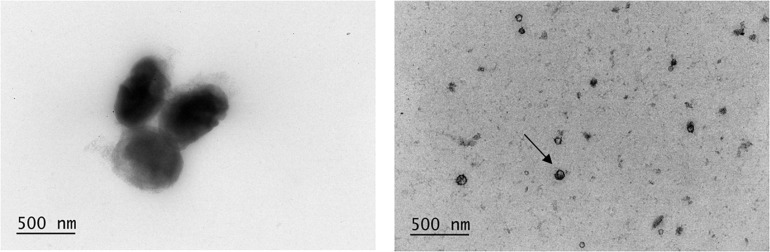
Electron micrographs of *O. splanchnicus* 57 cells and outer membrane vesicles (OMV, example pointed with an arrow).

Considering the potentially bioactive effector molecules secreted by *O. splanchnicus* 57, we studied the presence of OMVs in the bacterial culture. We used a previously published protocol (Elhenawy et al., 2014) with modifications to isolate OMVs. Transmission electron microscopy (TEM) was used to confirm the presence of OMVs in the preparations. Also, *O. splanchnicus* cells were also subjected to TEM. In the electron micrographs, *O. splanchnicus* 57 bacterial cells appeared as short rods as previously described in the literature (Göker et al., 2011). OMVs were clearly visible in the electron micrographs (Figure 3). The OMVs of *O. splanchnicus* 57 were observed to be smaller than 100 nm in size.

### Immunoregulatory Effect of *O. splanchnicus* 57 on HT-29 Cell Line

**FIGURE 4:**
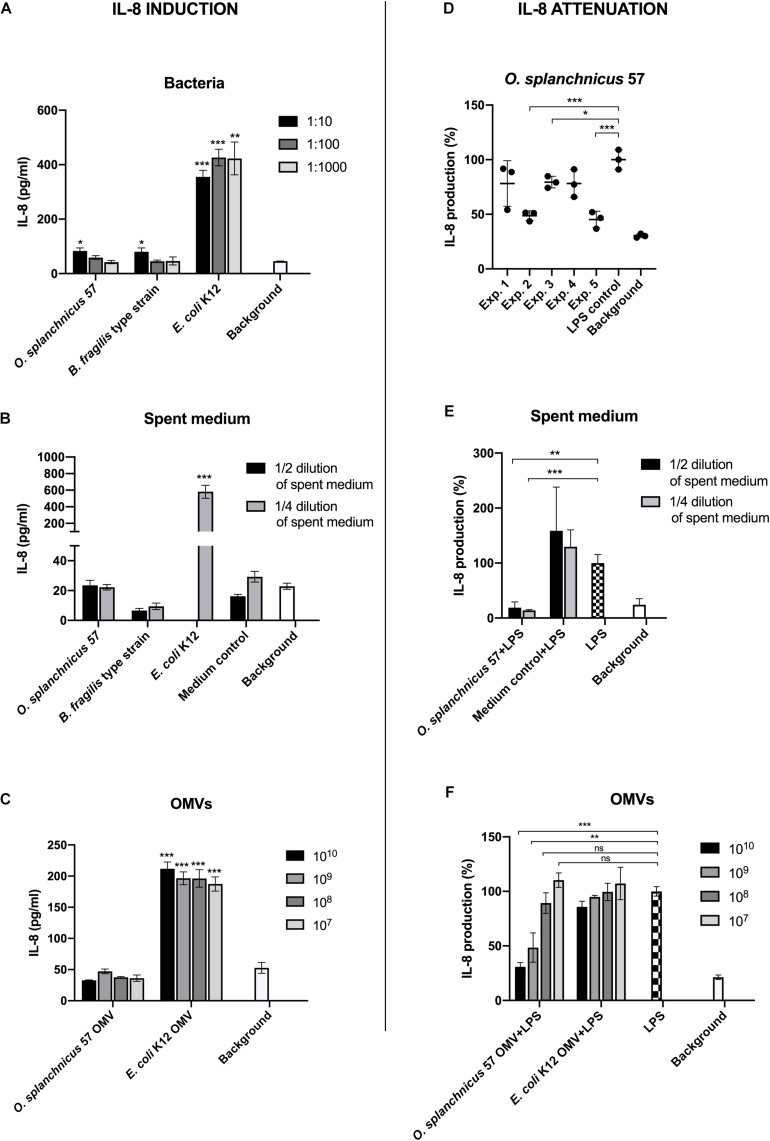
The induction of IL-8 production in HT-29 cells by *O. splanchnicus* 57 cells **(A)**, spent medium **(B)**, and OMVs **(C)**. Representative results are shown as a mean and standard deviation of three technical replicates. In the section B, the result of 1/2 dilution of *E. coli* K12 spent medium is not shown due to excessive toxicity to the HT-29 cells. The medium control in the spent media experiments refers to GAM medium. An asterisk indicates a significant (* = *p* < 0.05, ** = *p* < 0.01, *** = *p* < 0.001) IL-8 production above the background level. The anti-inflammatory capacity of *O. splanchnicus* 57 cells **(D)**, spent medium **(E)**, and OMVs **(F)** as assessed by a decrease in LPS-induced IL-8 production in HT-29 cells. In section D, experiment refers to an individual biological experiment using a new passage of HT-29 cells. Representative results are shown as a mean and standard deviation of three technical replicates. The growth medium for HT-29 cells, McCoy 5A, was used as background control in all experiments. The medium control in the spent media experiments refers to GAM medium. * = *p* < 0.05, ** = *p* < 0.01, *** = *p* < 0.001, ns = not significant.

Next, we assessed the effect of *O. splanchnicus* 57 cells, spent medium and OMVs on the induction of proinflammatory cytokine IL-8 in enterocytes. HT-29 cell line was used to assess the ability of the strain to attenuate LPS-induced inflammatory responses i.e., IL-8 release from enterocytes as Caco-2 cell line has defects in the TLR4 signaling, which causes unresponsiveness to LPS stimulation (Van De Walle et al., 2010). HT-29 cells were incubated with different concentrations of the bacterium, spent medium and vesicles (Figures 4A–C). *E. coli* K12 and its OMVs were used as proinflammatory controls and *B. fragilis* as a control that does not elicit a reaction in HT-29 cells. McCoy 5A medium, the growth medium for HT-29 cells, was included as the background control. The 1:10 dilution of *O. splanchnicus* 57 and *B. fragilis* cells (corresponding to 10^7^ cells per ml) prompted IL-8 responses that were above background but were four times lower as compared to the responses induced by *E. coli* K12 (Figure 4A). The 1:100 and 1:1000 dilutions of *O. splanchnicus* 57 and *B. fragilis* cells induced IL-8 release statistically at the similar level as the background showing a dose-dependent decrease in response, whereas *E. coli* K12 triggered a strong response with all the concentrations showing a saturated induction already with the most diluted sample. Thus, the difference in the proinflammatory capacity of *E. coli* and the two other commensals was clear. The same result could be observed when using spent medium, as both *O. splanchnicus* 57 and *B. fragilis* spent media diluted to 1/2 or 1/4 of the adjusted concentration elicited only a minor IL-8 response, which did not differ significantly from the background and GAM medium control (Figure 4B). In contrast, the 1/2 dilution of *E. coli* K12 spent medium was excessively toxic to the HT-29 cells (not shown due to partial cell death resulting in unreliable quantification), and 1/4 spent medium dilution triggered a major induction of IL-8 in HT-29 cells (582 ± 78 pg/ml) significantly above the background level. Also, OMVs isolated from *O. splanchnicus* 57 and *E. coli* K12 induced significantly distinct IL-8 responses with all the amounts used (10^7^ – 10^10^ vesicles) as *E. coli* K12 OMVs elicited a four-fold stronger response than *O. splanchnicus* 57 OMVs (Figure 4C).

Subsequently, we also examined the anti-inflammatory capacity of *O. splanchnicus* 57 by assessing whether the bacterium could attenuate *E. coli* LPS –induced IL-8 production in HT-29 cells (Figure 4D). In three biological experiments out of five, incubation with *O. splanchnicus* 57 with HT-29 monolayer prior to LPS-induced inflammation significantly decreased the IL-8 release. In the two other experiments, the IL-8 levels after *O. splanchnicus* 57 treatment were also lower than LPS control, but the difference did not reach statistical significance. The culture supernatant of *O. splanchnicus* 57 drastically attenuated the LPS-induced IL-8 response to the background level with both, 1/2 and 1/4, dilutions (Figure 4E). Medium control, i.e., GAM broth, did not have an effect as the induced IL-8 levels were the same or higher than the LPS control. Interestingly, also *O. splanchnicus* 57 OMVs dose-dependently decreased the IL-8 production in LPS-induced HT-29 cells suggesting an anti-inflammatory capacity (Figure 4F). The decrease was significant only with the two highest concentrations, 10^10^ and 10^9^ OMVs per well, which yielded a clear, 50% or more decrease as compared to the LPS control.

## Discussion

In the present study, we addressed the immunomodulatory properties of the relatively abundant, but poorly characterized *O. splanchnicus*. A recent study about distinct microbial niches in the colonic mucosa of twelve transplant donors showed that *Odoribacter* did not exhibit colonizing preference between cecum, transverse colon and sigmoid colon (James et al., 2020). The abundance of *Odoribacter* varied from undetectable to 1.6% of the total bacterial load, which corresponds to normal microbiota variability between individuals. Due to the lack of previous studies on the characteristics of this fairly abundant bacterium, we decided to address its immunomodulatory properties. The draft genome of *O. splanchnicus* 57 lacked the *lpxM* gene needed for hexa-acylation of lipid A, the moiety that determines the endotoxicity of LPS based on the ligand affinity to TRL4-MD2 complex (Park et al., 2009; Han et al., 2013). Thus, *O. splanchnicus* potentially harbors only penta-acylated lipid A, which is 100-fold less toxic compared to the *E. coli* LPS (Park et al., 2009). Hexa-acylated *E. coli* lipid A contains six acyl chains and two phosphate groups activating the TLR4–Mal–MyD88 pathway, whereas penta-acylated lipid A signals through TLR4–TRAM–TRIF causing lower cytokine release (Park et al., 2009). Bacteroidetes species are major contributors of the penta-acylated LPS biosynthesis in a healthy gut, as 80–90% of the total produced LPS is of Bacteroidetes origin (d’Hennezel et al., 2017). It has been suggested that the immunoinhibitory activity of gut microbial LPS load in the gut could facilitate host-tolerance to microbes due to the silencing of TLR4 signaling (d’Hennezel et al., 2017). The similar, low-level NF-**κ**B activity induced by *O. splanchnicus* 57 and penta-acylated *B. fragilis* LPS in the THP-1 reporter cell line support our previous hypothesis of *O. splanchnicus* 57 lipid A containing only five acyl chains (Weintraub et al., 1989). The LPS from *O. splanchnicus* 57 and *B. fragilis* also induced lower TNF-α/IL-10 ratios in human PBMCs compared to *E. coli* K12, but the effect was not statistically significant.

IL-10 is an essential anti-inflammatory cytokine for colon homeostasis and regulation of inflammation, whereas TNF-α mediates pro-inflammatory response (Kany et al., 2019). Recently, *O. splanchnicus* was found to correlate negatively with TNF-α production after LPS stimulation in a microbiome-cytokine interaction pattern study describing a connection between the abundance of a specific bacterial species in the gut and a high/low response of a certain cytokine (Schirmer et al., 2016). The microbiome could affect TNF-α production in a stimulus-dependent manner, as the microbial associations with TNF-α were only detected after LPS stimulation (Schirmer et al., 2016). There was no difference in our results regarding the effect of *O. splanchnicus* 57 cells and *E. coli* K12 on the ratio of TNF-α/IL-10 produced by human PBMCs. However, the spent medium of *O. splanchnicus* 57 did elicit a significant amount of IL-10 in relation to TNF-α, which indicated that the secreted bacterial molecules in the medium could have an anti-inflammatory impact on the host cells. A highly prevalent gut anaerobe, *Faecalibacterium prausnitzii*, and its culture supernatant decreased TNF-α and increased IL-10 secretion in mice with 2,4,6-trinitrobenzenesulphonic acid (TNBS)-induced colitis (Sokol et al., 2008). As *O. splanchnicus* is a known producer of SCFAs (Werner et al., 1975), these metabolites may have enhanced to some extent the upregulation of IL-10 production in PBMCs (Saemann et al., 2000; Cavaglieri et al., 2003; Chen et al., 2019). In a mouse colitis model, an induction of IL-10 upon FMT was observed, which was associated with the increased abundance of fecal SCFA-producing taxa, including *Odoribacter* (Burrello et al., 2018).

We found *O. splanchnicus* 57 cells to be non-adherent to HT-29 and Caco-2 cells or mucus. In addition, the bacterial cells did not have an effect on enterocyte monolayer integrity as measured by TER. These findings also insinuate that secreted molecules rather than the bacterial cells play a key role in the host-microbe interactions of *O. splanchnicus*. Many *Bacteroides* spp. have been shown *in vitro* to beneficially interact with enterocytes without cell-cell contact (Hiippala et al., 2020). Another well-studied example of closely related bacteria is *B. fragilis*, which produces an anti-inflammatory molecule, polysaccharide A (PSA), packed in OMVs and able to activate T-cell dependent immune responses in the host (Mazmanian et al., 2005; Shen et al., 2012). Besides SCFAs, very little is known about the effector molecules produced by *O. splanchnicus*. We hypothesized that *O. splanchnicus* 57 OMVs could transport beneficial effector molecules from the bacterium to the gut epithelium and tested this in an *in vitro* setting. We isolated and visualized *O. splanchnicus* 57 OMVs by TEM followed by assessing the anti-inflammatory capacity of the vesicles *in vitro*. *O. splanchnicus* 57 produced OMVs ubiquitously under normal, presumably non-stressed culture conditions.

Cytokine IL-8 is a neutrophil chemoattractant secreted by intestinal epithelial cells as a response to inflammatory stimuli, such as bacterial molecules (Lammers et al., 1994; Mitsuyama et al., 1994). Colonic IL-8 levels have been shown to correlate with the degree of intestinal inflammation and the number of neutrophils in the mucosa in UC and CD patients (Mitsuyama et al., 1994; Pearl et al., 2013). Undifferentiated HT-29 cells were used in the IL-8 assays due to the downregulation of IL-8 in LPS-induced differentiated HT-29 cells as well as due to the assay optimization in our previous studies using undifferentiated HT-29 cells (Böcker et al., 2000; Hiippala et al., 2016, 2020). However, gut epithelium consists of a cell population ranging from undifferentiated to fully differentiated enterocytes, and therefore bacterial anti-inflammatory activity should be studied also *in vivo*, *ex vivo* using tissue samples and/or gut organoid models to assess the response in an epithelium with enterocytes that have different maturation stages. First, we determined the IL-8 response in HT-29 cells induced by *O. splanchnicus* 57, its spent medium and OMVs. The bacterial cells or secreted molecules with different dilutions consistently triggered a cytokine production at a similar level as the background control or *B. fragilis*. Thus, *O. splanchnicus* 57 or its metabolites do not seem to provoke a substantial inflammatory reaction in the epithelium. Furthermore, *O. splanchnicus* 57 culture supernatant and OMVs clearly and consistently decreased the LPS-induced IL-8 release in HT-29 cells. Especially OMVs showed a dose-dependent attenuation capacity in enterocytes lowering the IL-8 response by over 50% with the two highest vesicle concentrations. To our knowledge, there are no previous studies on OMVs produced by *O. splanchnicus* and their immunoregulatory potential, which makes our findings novel in the field of gut microbiology.

Previously, the supernatant of *F. prausnitzii*, a highly abundant, butyrate-producing and anti-inflammatory bacterium in the healthy human intestinal mucosa and depleted in IBD patients (Sokol et al., 2008; Lopez-Siles et al., 2016; James et al., 2020), was found not to induce IL-8 secretion by Caco-2 cells, and on the other hand, to reduce IL-8 release from IL-1β-induced Caco-2 cells (Sokol et al., 2008). In addition, *F. prausnitzii* supernatant, but not the bacterial cells alone, strongly inhibited NF-κB activation by IL-1β in Caco-2 cells, which was not due to butyrate, as the effect could not be produced by SCFA (Sokol et al., 2008). More recently, the extracellular polymeric matrix and a specific 15 kDa protein have been found to contribute to the anti-inflammatory properties of *F. prausnitzii* (Rossi et al., 2015; Quevrain et al., 2016). Similarly, the effector molecules of *O. splanchnicus* need to be addressed in future studies. Our results with isolated OMVs from *O. splanchnicus* 57 show that the bacterium could exert anti-inflammatory and mucosal homeostasis-promoting effects in the gut, beyond the known beneficial health effects of SCFAs. We acknowledge that *in vitro* assays have limitations and should be considered as an early phase to explore the bacterium’s potential anti-inflammatory activity, which should be studied further by using more advanced *in vitro* and animal models.

Bacterial OMVs are known to carry a wide variety of bacterial molecules to the epithelium via the thick mucus layer that is physically inaccessible to bacterial cells and thus, to play a vital role in bacteria-host interactions (Lynch and Alegado, 2017). For example, an inflammation-silencing gut symbiont *Bacteroides vulgatus* has been shown to produce OMVs, which induce a tolerant semi-mature phenotype in CD11c + bone marrow-derived dendritic cells (Maerz et al., 2018). Furthermore, *A. muciniphila*-derived OMVs could attenuate the release of pro-inflammatory IL-6 from colon epithelial cells *in vitro* and decrease the severity of colitis in DSS-induced mouse model (Kang et al., 2013). OMVs produced by *A. muciniphila* also improved gut barrier integrity *in vitro* and metabolic functions *in vivo* (Chelakkot et al., 2018). Extracellular vesicles of *F. prausnitzii* upregulated anti-inflammatory cytokines and decreased proinflammatory cytokines *in vitro* (Jafari et al., 2019; Rabiei et al., 2019), whereas *F. prausnitzii* supernatant ameliorated colitis in mice models (Miquel et al., 2015; Huang et al., 2016). Concerning the possible effector molecules carried by OMVs, for example bacterial sphingolipids are also likely to be transported in OMVs through the mucus layer to the intestinal epithelium (Heaver et al., 2018). Sulfobacin B, a type of sulfonolipid produced by *O. splanchnicus*, suppressed LPS-induced inflammatory responses *in vitro* and *in vivo* in a murine model of colitis by inhibiting dose-dependently LPS-stimulated production of TNF-α in cultured mouse macrophages (Maeda et al., 2010).

In summary, we isolated *O. splanchnicus* strain 57 from a stool batch of healthy FMT donor, whose recipients were effectively treated for recurrent *Clostridioides difficile* infection. Our *in vitro* findings implicate *O. splanchnicus* as a non-adherent gut commensal with innocuous impact on the epithelial monolayer integrity. The genome data and *in vitro* assays implicate that *O. splanchnicus* possibly harbors a less toxic, penta-acylated LPS inducing similar effect as *B. fragilis*. The novel *O. splanchnicus* fecal isolate 57 produces OMVs under normal growth conditions as visualized by TEM. The bacterium, its culture supernatant or OMVs did not induce an IL-8 response in enterocytes indicating a low proinflammatory capacity. On the contrary, *O. splanchnicus* 57 and its effector molecules elicited *in vitro* anti-inflammatory action in enterocytes, as the bacterial cells, spent medium and OMVs decreased *E. coli* LSP-induced IL-8 production in HT-29 cells. The effect was especially robust with *O. splanchnicus* culture supernatant and OMVs, indicating that the bacterium’s epithelial interactions do not require cell-cell contact. The beneficial immunoregulatory properties of secreted bacterial molecules and their impact on gut homeostasis is an exciting, thus far relatively unexplored domain in the microbiome research. Further studies are warranted to identify the potential inflammation-ameliorating, OMV-transported effector molecules of *O. splanchnicus.*

## Materials and Methods

### Isolation and Identification of *O. splanchnicus* Isolate 57

*Odoribacter splanchnicus* isolate 57 was isolated from the feces of a healthy, pre-screened FMT donor as described in our previous study (Hiippala et al., 2020). The use of the donor sample was approved by the Ethics Committee of Hospital District of Helsinki and Uusimaa Finland (DnroHUS124/13/03/01/11). The donor, described in a previous study (Jalanka et al., 2016), provided a written informed consent. In short, the frozen banked fecal solution (saline-10% glycerol) was thawed, serially diluted in PBS and cultivated on Gifu anaerobic medium (GAM; Nissui Pharmaceutical Co., Ltd., Japan) for 48 h under anaerobic conditions (Concept Plus anaerobic workstation, Ruskinn Technology Ltd., Bridgend, United Kingdom) at 37°C. Separate colonies were picked and re-streaked on new agar plates until they were purified. Gram-staining and microscopy were used to evaluate the purity of the isolate followed by partial 16S rRNA gene sequencing for tentative identification. Bacterial mass from the isolate was resuspended in TE buffer (10 mM Tris–HCl, 1 mM EDTA, pH 8), heated to 95°C for 15 min to break the cells and used as PCR template. PCR amplification of the partial 16S rRNA gene was carried out using 27F DegL (5′-AGR GTT YGA TYM TGG CTC AG-3′) forward and Pd (5′-GTA TTA CCG CGG CTG CTG-3′) reverse primers. Sanger sequencing of the PCR product using the forward primer was carried out in the Institute of Biotechnology core facility, University of Helsinki. The partial 16S rRNA gene sequence was compared to NCBI 16S ribosomal RNA sequences database using BLASTn to acquire a genus-level identification.

### Genome Sequencing

Genomic DNA was extracted from *O. splanchnicus* 57 cell pellet with DNeasy Blood and Tissue Kit (Qiagen, Hilden, Germany) following the manufacturer’s protocol for Gram-negative bacteria and fragmented to approximately 500 bp using a Q800R2 Sonicator (QSonica, Newtown, CT, United States). Genome libraries were prepared for paired end sequencing using the NEBNext^®^ Ultra^TM^ II Kit (New England Biolabs, Ipswich, MA, United States) and quantification was carried out using the Library Quantification Kit (KAPA Biosystems, Waltham, MA, United States). Libraries were pooled in equimolar amounts and sequenced on the MiSeq (Illumina, Inc., San Diego, CA, United States). Short read data were assembled using unmanned genome assembly pipeline (UGAP;^1^) using the SPAdes genome assembler. The species of each genome was identified with Kraken (Wood and Salzberg, 2014). Read data were deposited in the NCBI SRA under BioProject PRJNA575760. Genomic comparison to *O. splanchnicus* type strain 1651/6^*T*^ genome (NC_015160.1) and screening of the genes that encode enzymes for Kdo2-lipid A modification we carried out using BLAST (Basic Local Alignment Search Tool, BLASTn algorithm).

### Other Bacterial Strains and Growth Conditions

*Bacteroides fragilis* type strain E-022248T (= DSM 2151 = ATCC 25285) from the VTT Culture Collection (VTT Technical Research Center of Finland) was grown under anaerobic conditions for 2 days at 37°C on Brucella agar with hemin and vitamin K (Sigma Aldrich, St. Louis, MO, United States) along with 5% defibrinated sheep blood (Bio Karjalohja Oy, Finland). *Escherichia coli* K12-derived TOP10 (Invitrogen, United States) was aerobically cultivated overnight in Luria–Bertani broth (Becton Dickinson, United States).

### Epithelial Cell Lines

The human colonic epithelial cell lines Caco-2 (ACC 169) and HT-29 (ACC 299) were obtained from the German Collection of Microorganisms and Cell Cultures (DSMZ). Cell lines were grown at 37°C in an incubator under an oxic atmosphere with 5% CO_2_ and passaged after reaching 80% confluence (approximately every 3–4 days) using TryplExpress (Lonza, United States) to detach the cells. Passages 6–28 were used in the experiments. HT-29 cells were cultivated in McCoy 5A (Lonza, Belgium) medium containing 10% heat-inactivated (30 min at 56°C) fetal bovine serum (FBS; Gibco) and 100 U ml^–1^ PEST. Caco-2 cells were grown in RPMI 1640 medium (Sigma-Aldrich, United States) supplemented with 20% FBS, non-essential amino acids (1%, NEAA; Lonza, Belgium), 15 mM HEPES (Lonza, Belgium), 100 U ml-1 penicillin and streptomycin (PEST; Lonza, Belgium) and 2 mM L-glutamine (Lonza, Belgium). Human THP-1 dual reporter monocytic cell line (thpd-nfis, Invivogen, United States) were cultured at 1 × 10^5^ cells/ml in 96-well plates in RPMI 1640 (Gibco, United States) supplemented with 10% fetal bovine serum (Gibco) and 100 U ml^–1^ penicillin and streptomycin.

### Adhesion to HT-29, Caco-2 and Mucus

The adherence of *O. splanchnicus* isolate 57 to Caco-2 and HT-29 cell lines (8 days post-plating) and mucus was studied as previously described (Kainulainen et al., 2013; Reunanen et al., 2015). The bacterium was grown in GAM medium supplemented with 10 μl ml^–1^ of [6′-^3^H]thymidine (17,6 Ci mmol^–1^, Perkin Elmer, United States) to metabolically radiolabel the cells. Six technical replicates (parallel wells) were used in each experiment. To assess the adherence to intestinal mucus, porcine mucus (Sigma-Aldrich, 50 μg well-1 in PBS) was allowed to absorb to Maxisorp microtiter plate wells overnight at 4°C. 10,000 Caco-2 or HT-29 cells per well were seeded onto 96-well microplate. [^3^H]Thymidine-labeled *O. splanchnicus* isolate 57 cells were washed with an appropriate medium (McCoy 5A for HT-29 cells, RPMI 1640 for Caco-2 cells and PBS for the mucus assay) without the supplements and adjusted to OD_600__*nm*_ 0.25 corresponding to approximately 10^8^ cells/ml. After 1 h of incubation on the epithelial cell monolayer or mucus at 37°C in a CO_2_ incubator, the bacterial suspensions were removed, and wells were washed three times to remove the non-adherent bacteria. Adhered bacteria were lysed with 1% SDS-0.1 M NaOH solution and radioactivity was measured with a liquid scintillator (Wallac Winspectral 1414, Perkin-Elmer, Waltham, MA, United States). The percentage of bound bacteria was calculated relative to the radioactivity of the bacterial suspension initially added to the wells.

### Effect on Caco-2 Monolayer Integrity

Caco-2 cell line is suitable for the monolayer integrity studies measuring TER due to the cells’ enterocytic differentiation and expression of intercellular junctional complexes (Hidalgo et al., 1989; Klingberg et al., 2005; Hsieh et al., 2015). The effect of *O. splanchnicus* 57 isolate on the TER of Caco-2 monolayer was studied as previously described (Kainulainen et al., 2015; Reunanen et al., 2015). Briefly, 50,000 Caco-2 cells were seeded on PET inserts with a pore size of 3 μm (Sarstedt, Nümbrecht, Germany) and grown for 8 days. The medium in the well was changed every 3 days. *E. coli* TOP10 was used as a negative control in the experiments due to its detrimental effect on monolayer integrity (Reunanen et al., 2015). *O. splanchnicus* 57 was washed with RPMI medium including the supplements, adjusted to OD_600__*nm*_ 0.25 and 100 μl of bacterial suspension was added onto cell monolayer. The TER was measured after 0 and 24 h using an EVOM epithelial voltmeter with an electrode (World Precision Instruments, United Kingdom). The blank resistance (measurement at time point 0) was subtracted from the measurements made after 24 h of incubation, and the unit area resistance (Ω cm^2^) was calculated by multiplying the tissue resistance values by the surface area of the filter membrane. The change in TER during the 24 h was calculated comparing the samples to the medium control.

### LPS Purification by the Chloroform-Methanol Method

*Odoribacter splanchnicus* 57, *B. fragilis* and *E. coli* K12 were grown in an appropriate culture broth. Cells were harvested by centrifugation (4,000 rpm, 10 min) and resuspended in single-phase Bligh and Dyer mixture consisting of chloroform/methanol/water (1:2:0.8 v/v) to remove phospholipids (Vatanen et al., 2016). After 20 min of incubation at room temperature, the suspension was centrifuged, and the cell pellet was washed twice with Bligh and Dyer mixture and the remaining LPS preparation was suspended in PBS. The yielded LPS concentration was quantified using the chromogenic LAL assay (Thermofisher, United States), by following the manufacturer’s instructions.

### LPS-Induced Activation of NF-κB in THP-1 Cell Line

Following overnight incubation, THP-1 dual reporter cells were induced to M0 macrophage differentiation with 15 ng/ml PMA for 24 h. Upon differentiation, cells were stimulated for 24 h with 10 ng/ml LPS from different bacterial strains: *O. splanchnicus* 57; *B. fragilis* type strain; *E. coli* K12 (tlrl-eklps InvivoGen; United States), smooth LPS from *E. coli* 0111:B4 (tlrl-eblps, Invivogen; United States), semi-rought LPS from *E. coli* R515 and rough LPS from *E. coli* J5 (IAX-100-007-M001 and 014-M001, respectively, Adipogen; United States). PBS was used as a control. Following 24-h stimulation, the supernatant was collected for the measurement of NF-**κ**B activity.

THP-1 dual cell line NF-κB activity was measured according to the manufacturer’s instructions (InvivoGen; United States). Briefly, the reporter cells have a stable integrator secreted alkaline phosphatase (SEAP) reporter gene for monitoring nuclear factor (NF)-κB activation. Upon NF-κB activation, the SEAP reporter gene is activated, leading to the secretion of alkaline phosphatase, which is then quantifiable by a colorimetric assay (QUANTI-Blue, InvivoGen; United States) using a microplate reader (FluoStar Omega, BMG Labtech; Germany) with absorbance set for 630 nm.

### Peripheral Blood Mononuclear Cell Isolation

Buffy coat blood from healthy donors were obtained from the IRCCS Policlinico San Matteo, Pavia, Italy. PBMCs were isolated using density gradient centrifugation (Ficoll-Paque Plus, GE Healthcare) according to the manufacturer’s instructions. Briefly, blood sample was diluted with DPBS and layered on Ficoll-Paque Plus. After centrifugation (300 g, 30 min), the separated mononuclear cells were collected, resuspended in DPBS and washed 3 times with DBPS to remove the platelets. Cells were resuspended in DPBS + 0.5% BSA + 2mM EDTA and counted.

### TNF-α/IL-10 Released From Human PBMCs After Co-Incubation With *Odoribacter* LPS, *Odoribacter* Cells and Spent Medium

PBMCs were added to 48-well plates (5,00,000 cells per well) in DMEM medium (ThermoFisher Sci.) supplemented with 10% FBS. *O. splanchnicus* 57, *B. fragilis* and *E. coli* K12 LPS preparations were diluted to 1 ng/ml in DMEM + 10% FBS. *O. splanchnicus* 57 was grown in GAM broth under anaerobic conditions for 2 days. The bacterial culture was centrifuged (10, 000 rpm, 3 min) and the cell pellet was washed with DMEM + 10% FBS, adjusted to OD_600__*nm*_ 0.25 and 50 μl of this suspension was added onto PBMCs. DMEM + 10% FBS was used as background control. For the experiments with *O. splanchnicus* 57 spent medium, the bacterial suspension was adjusted to OD_600__*nm*_ 1.0, centrifuged (16 000 rpm, 3 min) and the supernatant was filtered with 0.2 μm filter. 40 μl of the spent medium preparation (5%) was added onto PBMCs. Heat-killed *E. coli* K12 MG1655 (10^8^ cells/ml) and the bacterial growth medium (GAM) were used as controls in the experiments with bacterial cells and spent medium. PBMCs were incubated with LPS, bacterial cells or spent medium for 24 h and the supernatants were collected for cytokine analysis. TNF-α and IL-10 (hIL-10 JES3-19F1 clone; hTNF-a MAb11 clone, Biolegend, United States) were measured by using ELISA assay according to the manufacturer’s instructions. Unstimulated PBMCs were used as control in all experiments. In the LPS stimulation, the results were calculated as a ratio of TNF-α/IL-10 using PBMCs isolated from the buffy coats of four different donors. In the experiment of *O. splanchnicus* 57 spent medium and bacterial cell stimulation, the TNF-α/IL-10 ratio was calculated as a mean and standard deviation of three or four experiments by using PMBCs isolated from buffy coats of three or four donors, respectively.

### OMV Isolation

Outer membrane vesicles isolation from 400 ml of *O. splanchnicus* 57 and *E. coli* K12 broth culture was carried out as previously described (Elhenawy et al., 2014) with modifications by pelleting the bacteria (centrifugation 4000 rpm for 10 min), filtering the supernatant with 0.2 μm filter to remove any remaining bacteria and concentrating the filtered supernatant by using Amicon 100 kDa filters (Millipore). The concentrated supernatant was ultracentrifuged (Beckman Coulter) for 2 h at 32,000 rpm. The remaining OMV pellet was resuspended in PBS and ultracentrifuged (2 h, 32,000 rpm) again. The isolated OMVs were suspended in PBS for nanoparticle tracking analysis (NTA) for quantification and electron microscopy (EM).

### Sample Preparation and Transmission Electron Microscopy

OMVs and bacterial samples were prepared for electron microscopy (EM) by loading to carbon coated and glow discharged 200 mesh copper grids with pioloform support membrane (Puhka et al., 2017). Samples were fixed with 2.0% PFA in NaPO4 buffer, stained with 2% neutral uranyl acetate, further stained and embedded in uranyl acetate and methyl cellulose mixture (1.8/0.4%). OMVs and bacterial cells were viewed with transmission EM using Jeol JEM-1400 (Jeol Ltd., Tokyo, Japan) operating at 80 kV. Images were taken with Gatan Orius SC 1000B CCD-camera (Gatan Inc., United States) with 4008 × 2672 px image size and no binning.

### The Induction of IL-8 Production in HT-29 Cells

The measurement of IL-8 response in 8 days post-plating HT-29 cells by bacterial cells, spent medium and OMVs was carried out as previously described (Kainulainen et al., 2013). In brief, the bacterial suspensions were washed with McCoy 5A medium supplemented with 10% FBS and adjusted to OD_600__*nm*_ 0.25. Bacteria were diluted to 1:10, 1:100, and 1:1,000 and incubated on the HT-29 cells for 3 h at 37°C in a CO_2_ incubator. Spent media from broth cultures of *O. splanchnicus* 57, *B. fragilis* type strain and *E. coli* K12 were prepared by adjusting the bacterial culture to OD_600__*nm*_ 1.0, pelleting the bacteria by centrifugation and filtering the supernatant through 0.2 μm filter to remove any remaining cells. Spent medium preparation was diluted to 1:4 and 1:2 using McCoy 5A medium with supplements. Bacterial growth medium (GAM) diluted with McCoy 5A medium was used as control. OMVs were diluted to McCoy 5A with supplements and added on monolayers using 10^10^, 10^9^, 10^8^, and 10^7^ concentrations. Three technical replicates (parallel wells) were used in each experiment. OptEIA Human IL-8 ELISA kit (BD Biosciences, United States) was used according to the manufacturer’s instructions to measure the concentration of the chemokine in the culture media.

### Anti-Inflammatory Capacity

IL-8 assays were only performed using the HT-29 cell line, because Caco-2 cells are known to be unresponsive to LPS stimulation possibly due to defects in TLR4 signaling (Funda et al., 2001; Van De Walle et al., 2010; Hsu et al., 2011). The capacity of *O. splanchnicus* 57 isolate or its effector molecules to attenuate LPS-induced IL-8 production in HT-29 cells was measured as previously described (Kainulainen et al., 2015; Hiippala et al., 2020). In brief, HT-29 cells were seeded 10,000 cells per well onto 96-well microplates for the attenuation assay. Broth culture of *O. splanchnicus* 57 was washed once with McCoy 5A medium supplemented with FBS (10%) and adjusted to OD_600__*nm*_ 0.25 using the same medium. 100 μl of bacterial suspension was added onto 8 days old HT-29 cells and incubated at 37°C for 1 h under oxic atmosphere with 5% CO_2_. Spent medium and OMV dilutions were prepared as in the experiments measuring IL-8 release. McCoy 5A medium containing 10% FBS without LPS was used as the background control. In the experiments with spent medium, GAM broth with LPS was used as medium control. Next, the bacterial suspension, spent medium or OMVs were removed from the HT-29 monolayer and 200 μl McCoy 5A medium with 1 ng/ml of *E. coli* LPS (Sigma) was added. After a 4-h incubation with LPS, supernatants were collected and IL-8 levels were measured by using an ELISA assay (BD OptEIATM Set). The IL-8 levels were calculated using four parametric logistic curves. The attenuation capacity of the isolate, spent medium and OMVs were assessed by comparing the LPS-induced IL-8 production (%) of the sample to that of the LPS control (100%). The experiment with *O. splanchnicus* 57 bacterial cells was repeated five times.

### Statistical Testing

All the experiments were repeated two to five times (biological replicates) depending on the assay to confirm the results. An unpaired *t*-test was used to determine significant differences between two groups, such as the sample and the control. Homoscedasticity testing was performed with Levene’s test to identify equal or unequal variances. All statistical analyses were carried out with GraphPad Prism 8.4.1 (GraphPad Software, United States). A *p*-value of <0.05 was considered statistically significant.

## Data Availability Statement

The datasets presented in this study can be found in online repositories. The names of the repository/repositories and accession number(s) can be found below: https://www.ncbi.nlm.nih.gov/, PRJNA575760.

## Ethics Statement

The studies involving human participants were reviewed and approved by the Ethics Committee of Hospital District of Helsinki and Uusimaa Finland (DnroHUS124/13/03/01/11). The patients/participants provided their written informed consent to participate in this study.

## Author Contributions

RS and KH conceptualization. RS, KH, GB, CB, FF, VK, and JB methodology. KH, GB, CB, AD-B, MS, JB, and VK validation. KH, DL, and JB formal analysis. KH and RS writing—original draft preparation. KH and GB writing—review and editing, all authors, and visualization. RS, FF, KE, and DE supervision. RS, KH, and VK funding acquisition. All authors have read and agreed to the published version of the manuscript.

## Conflict of Interest

The authors declare that the research was conducted in the absence of any commercial or financial relationships that could be construed as a potential conflict of interest.

## Footnotes

1 https://github.com/jasonsahl/UGAP
