# Supplementary material for: Novel Odoribacter splanchnicus Strain and Its Outer Membrane Vesicles Exert Immunoregulatory Effects in vitro
Source: Front Microbiol. 2020 Nov 12;11:575455. doi: 10.3389/fmicb.2020.575455 (PMC7689251; doi:10.3389/fmicb.2020.575455)
Supplement: Supplementary file 1 [file Data_Sheet_1.pdf]

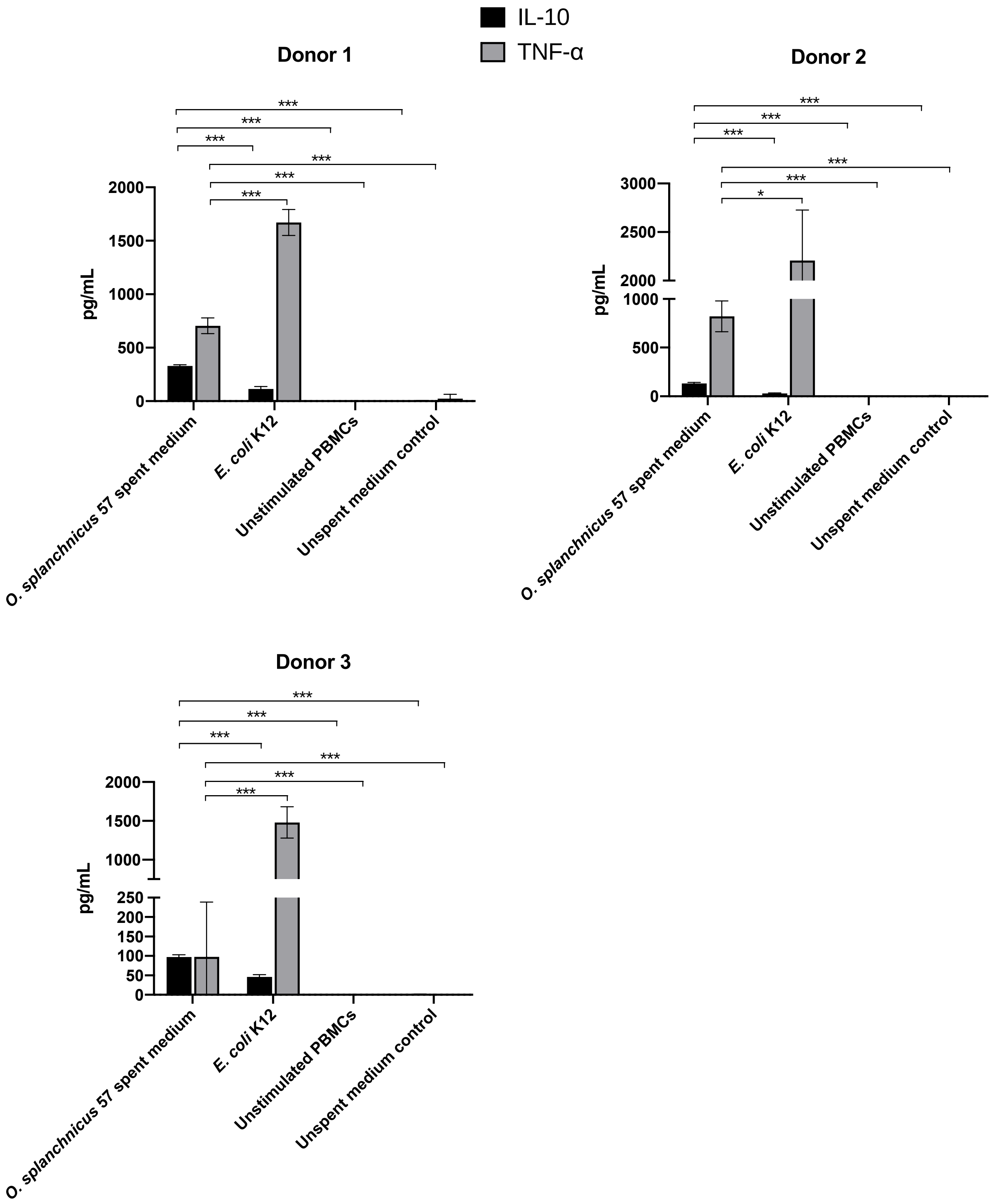

SUPPLEMENTARY FIGURE 1. Total concentrations of TNF-α and IL-10 released from PBMCs of three different donors after stimulation with *O. splanchnicus* 57 spent medium. Heat-killed *E. coli* K12, unstimulated PBMCs and unspent medium were used as controls.

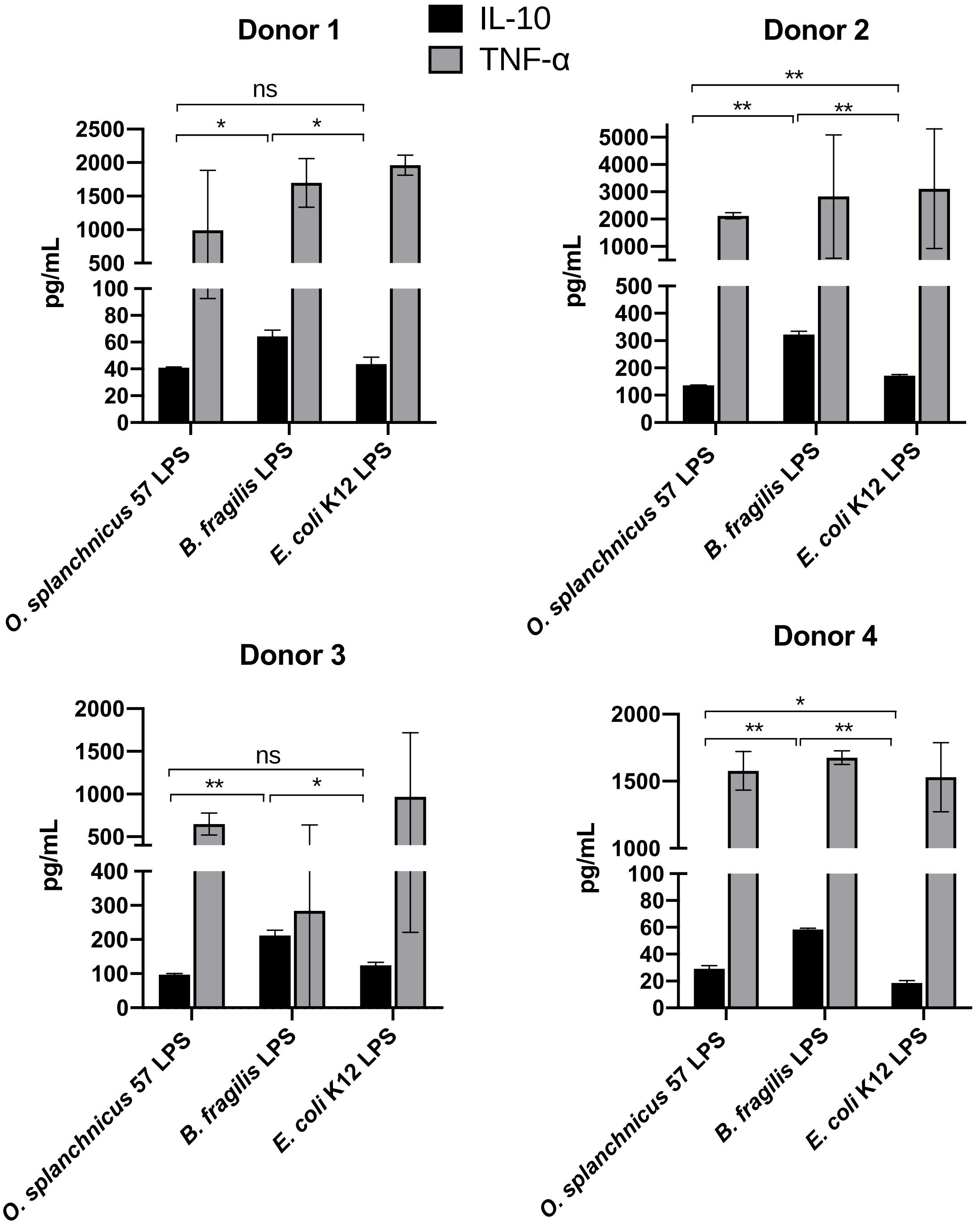

SUPPLEMENTARY FIGURE 2. Total concentrations of TNF-α and IL-10 released from PBMCs of four different donors after stimulation with *O. splanchnicus* 57 LPS, *B. fragilis* type strain LPS and *E. coli* K12 LPS.
